# Supplementary figures and images for: Comparative analyses of parasites with a comprehensive database of genome-scale metabolic models
Source: PLoS Comput Biol. 2022 Feb 23;18(2):e1009870. doi: 10.1371/journal.pcbi.1009870 (PMC8901074; doi:10.1371/journal.pcbi.1009870)

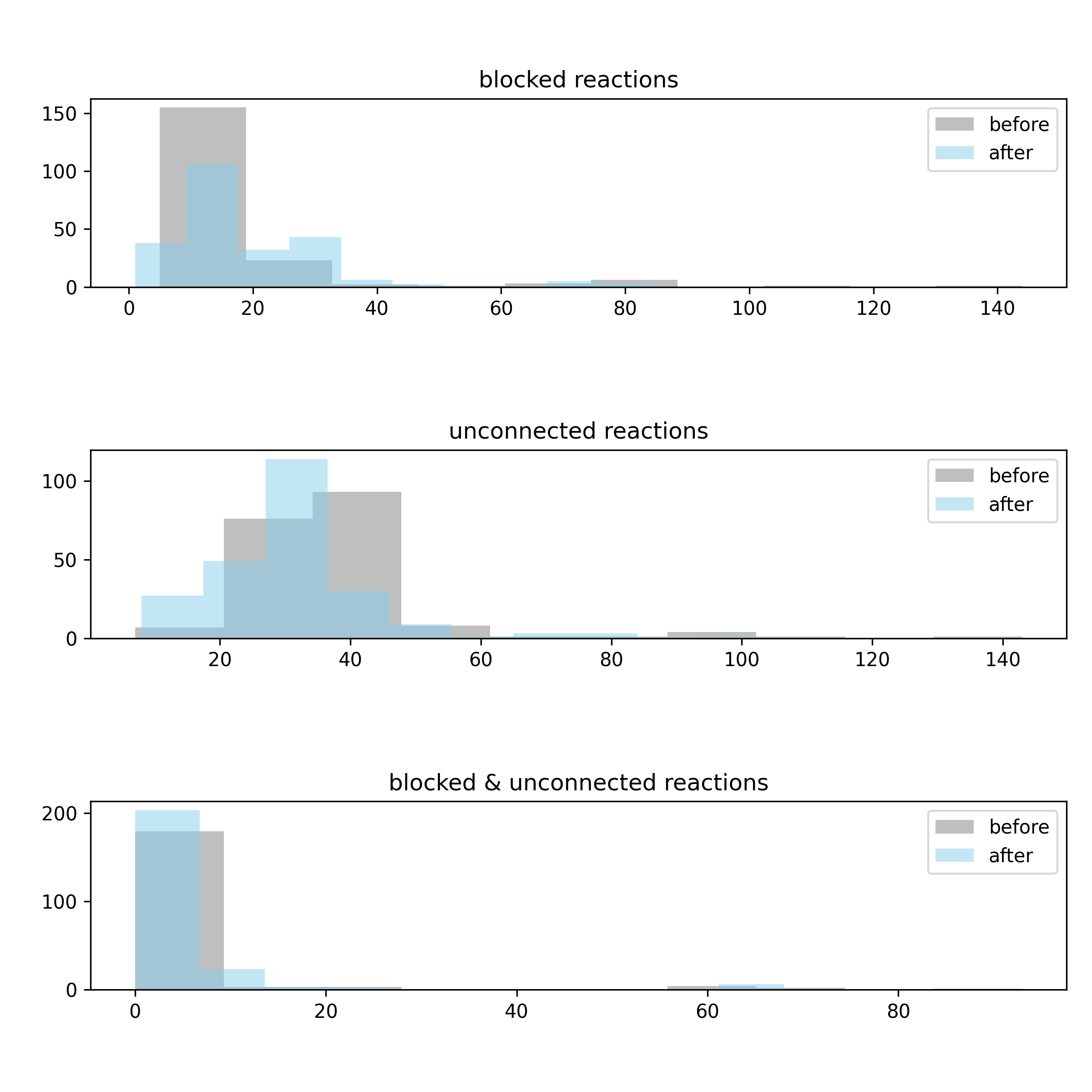

Supplement: S9 Fig — The distribution of poorly connected reactions (A: blocked, B: unconnected, C: both) was similar before and after gapfilling. (TIFF) [file pcbi.1009870.s015.tiff]
