# Supplementary material for: Comparative analyses of parasites with a comprehensive database of genome-scale metabolic models
Source: PLoS Comput Biol. 2022 Feb 23;18(2):e1009870. doi: 10.1371/journal.pcbi.1009870 (PMC8901074; doi:10.1371/journal.pcbi.1009870)
Supplement: Attachment — Submitted filename: response_to_reviewers.docx [file pcbi.1009870.s016.docx]

Thank you to the editor and reviewers for thoughtful assessments of our work. Below we have copied the reviewer comments (in blue) and addressed each below (in black) and in the manuscript. Please note, all page and line numbers provided only refer to the ‘clean’ manuscript, not to the ‘marked up’ manuscript.

Reviewer #1: I congratulate the authors for creating a very important knowledge-base to the parasite community. The paper is very well written, the methods used are solid and the results reported are of extreme importance for the scientific community. I especially appreciate that the authors propose that the method will be used iteratively and models can be improved as more data arise.

As I mention in the revision below tha I believe that more and more the scientific community should be careful when only propagating data from closely related species. And the authors indeed show in their results that "Phylogeny is not the sole predictor of model similarity". This idea can also be extended to the genomic prediction, annotation, metabolic models reconstruction, and so on and we should keep in mind that much information might be lost when we try to tailor-made the models based mostly on related species.

This is also why I believe that creating this extremely rich and diverse knowledge-base becomes of utter importance to future advances in the area.

Thank you!

Comments and questions:

1. You describe that reconstructions were built based on the annotated genomes, and then proteins were re-annotated using Diamond against the BiGG database.
However, I would like to know and you could also discuss this within the paper about the genomic annotation present within EuPathDB, as I don't imagine that all annotations are done in a homogeneous way across distinct databases.
And if this is correct, how do you handle these annotations coming from different sources? Could you discuss in terms of how robust is your pipeline taking into account this kind of differences?

You are accurate in assuming that each database may use different techniques to annotate genomes. In addition, ‘manual’ efforts such as protein biochemical characterization informs some gene annotations, but this has certainly not been attempted for all genes and all genomes. We added a discussion of this on page 16, lines 32-34. Our pipeline ‘controls’ for these differences by performing Diamond-based annotations for all genomes in addition to using EuPathDB’s annotations.

It would be interesting to also include for each genome within your dataset the proportion of uncharacterized protein coding genes or genes having DUFs (domains of unknown function) and whether these correlate with the proportion of reactions that are gap-filled in the final step.

Uncharacterized genes are handled in different ways in each database. This is referenced in the Table 1 caption. In brief, we quantified ‘uncharacterized’ genes in Table 1 (formerly Figure 1A) by searching EuPathDB searches for terms such as ‘uncharacterized’, ‘putative’, and ‘hypothetical’ for a representative strain. Because each database is heavily influenced by the respective scientific community, some databases do not use these terms. For example, these terms are not used on CryptoDB because the function of so few genes have been validated in the *Cryptosporidium* parasites (<20). Thus, the genomes of the *Cryptosporidium* parasites are mostly hypothetical and proposed functions are only putative or unknown (i.e. *C. parvum* Iowa II gene identifier: cgd3_3040, a cAMP-dependent protein kinase). In contrast, PlasmoDB regularly uses these terms because the biochemical and genetic assays to validate gene function have been much more well established and, thus, many functions are validated. Thus, we don’t find this quantification of uncharacterized protein coding genes to be very helpful and present it with this caveat.

2. In terms of compartmentalization, which was the proportion of transport reactions included in the models in order to allow for correct fluxes of reactions within compartments and how many of these are orphan reactions?

On average, 53% of transporters included in our reconstructions are genetically supported (see distribution below left). Additionally, 68% of transporters move metabolites between intracellular compartments rather than from the extracellular compartment into the cell (see distribution below right). These figures have been added as Supplemental Figure 8.

Is this one the reasons you did not include compartments for all species or were there other reasons for this choice?

We did not include compartments for all species as a practical choice, not biological choice. Many parasites have unusual compartments (and can vary by database and within database). We did not want to include any errors by accidentally propagating the wrong compartments, so we only included extensive compartmentalization for those that we had high confidence (documented in Supplemental Table 5).

3. In page 4 (lines 7 to 11) and in Supp. Fig 1: I am not sure if I understood Suppl Figure 1C and D correctly. Are these the categories of genes that are only in EuPathDB (C) or these are all genes in EuPathDB?

This figure caption has been clarified (page 6). In brief, these genes are on EuPathDB and the reconstruction of interest that are associated with individual pathways (so the intersection shown in Supplemental Figure 1A for three individual pathways). The figure shows the consistency between EuPathDB and our reconstructions.

Also, regarding these genes that were not included in the reconstructions and by referring also to the methods described on page 17 lines 23-40: what is the distribution of genes belonging to each of the 3 groups you mention? And do these percentages change depending on the database used (for example TritrypDB versus ToxoDB)?

The distribution of genes in each of the three sample pathways is shown in Suppl. Figure 1C (reproduced below). To look at this on a database-by-database level, we have broken these data down by phylogenetic/database grouping (see figures below).

Supplemental Figure 1:

Distribution of genes in each pathway by database (breaking down Suppl. Figure 1C by database):

Coverage of EuPathDB genes associated with each pathway in the reconstructions, broken down by database (breaking down Suppl. Figure 1D by database):

4. You mention that you perform a brief manual curation of models. I would like to know time-wise how long is brief in this case for comparison reasons to fully curation that can take up much time. This is a minor comment, but I think it would be important to state in the text whether the authors are planning to perform this type of curation with other species. This could be discussed as a perspective as it would be of great interest to the broader parasite community to have models that were constructed similarly in order to be comparable within different taxa.

We have included a discussion that manual curation will be important for all of these reconstructions on page 13 lines 2-4, 9-12, & 28-29, page 14 lines 17-19, and page 15 lines 10-13. For *Plasmodium falciparum* 3D7 specifically, we described all modifications on page 17 lines 14-20.

5. Can you describe a little more the reactions that were present in the manual curation that were not added to the final semi-curated Plasmodium models? Why do you think these are not included and is it possible to propose a way to improve this step of semi-curation in the future in order to arrive at models closer to curated ones?

Based on our manually inspection of the genes (and therefore reactions) that were omitted from the semi-curation models but present in the manual curation, we have categorized them into several groups:

- group 1: non-specific genes that map to multiple reactions
  - e.g. PF3D7_0103200 encodes a nucleoside transporter so there is no single KEGG or BiGG reaction ID that corresponds with its function.
- group 2: tRNAs
  - e.g. PF3D7_1341000 encodes the tRNA for arginine, which is not an enzyme but is included in the curated reconstruction
- group 3: metabolic functions not yet covered by the BiGG database but were made specifically for the manually curated reconstruction
  - e.g. PF3D7_0919600 encodes a dolichyl-diphosphooligosaccharide-protein glycotransferase
- group 4: metabolic functions already in draft model, but can be catalyzed by multiple genes
  - e.g. PF3D7_0211800 encodes asparagine--tRNA ligase, reaction was not added to reaction X because it already had gene X associated with it

These genes and reactions should be the first focus of manual curation efforts; this has been highlighted on page 13 lines 24-29. Group 1 and 2 genes are likely the easiest annotations to add, whereas group 3 genes require more careful attention and custom reaction construction. In future iterations of the pipeline, we will add group 4 genes.

6. In Figure 3: You could use the colors of databases provided in 3A and 3C on the individual points for Figure 3B.

We have made this modification per the reviewer’s suggestion.

Also, it seems that not only number of reactions but also number of unique reactions are related to genome size, correct? It would be interesting to see the same trend as showed in Figure 3B but also taking into account unique reactions, especially since your barplots in 3C do not account for per genome variability.

The number of unique reactions is statistically correlated with the genome size, as shown below. This figure has been added to Supplemental Figure 4 and is referenced page 7 lines 10-11.

I would also highlight the genomes that actually deviate from this linear regression curve in order to pinpoint whether these differences arise from biological traits or if they are due to technical differences.

These deviant points are generally organisms that are phylogenetically more unique, such as *T. vaginalis* G3 (the only genome on TrichDB) or *C. velia* CCMP2878 and *V. brassicaformis* CCMP3155 (two of the three non-*Cryptosporidium* species on CryptoDB).

7. Still in Figure 3 (and the text): Could you explain why you don't find basic pathways such as glycolysis, TCA cycle, Pentose Phosphate, etc within your core reactions? It would be interesting to know in which models these reactions are not taking place and not only knowing they take place in ~90% of the models. Can you also explain if this is merely a technical issue of if this is in accordance with the biology of these species lacking central metabolism enzymes?

It is known that parasite have reduced canonical core metabolism. For example, *Plasmodium* species do not use the TCA cycle and oxidative phosphorylation for energy production and some *Cryptosporidium* species lack TCA cycle enzymes entirely.

8. In page 5, line 33 you mention that even small reconstructions contain unique reactions prior to gapfilling. I am also wonder if these numbers change a lot after the gapfilling process.

Following gapfilling, the relationship between the number of unique reactions is weakened (see below); however, it remains true that even small reconstructions contain unique reactions after gapfilling. These figures have been added to Supplemental Figure 4 and referenced on page 7 lines 10-11.

Before gapfilling (Suppl. Fig. 4F):	After gapfilling (Suppl. Fig. 4G):

9. I am not sure if there is a particular reason the authors do not discuss or compare also previous databases of metabolic networks such as Metacyc. I would at least advise you to explain if you perform better and if possible add an explanation as to why or state if these are not comparable instances of models from the same species.

In the revised manuscript, we have added improved referenced to MetaCyc (see page 4 line 14). However, MetaCyc does not include as many protozoan parasites as ParaDIGM; for example, ParaDIGM contains 12 *Toxoplasma* genomes whereas MetaCyc has only one (*T. gondii* ME49). Additionally, to our knowledge, MetaCyc does not contain curated metabolic data for any of the species that we have modeled and, thus, we chose to compare our reconstructions to only manually curated published reconstructions.

Also this is of interest since as you mention in page 11 line 3, reactions not included in BiGG database are not within your set of universal reactions.

No database is completely comprehensive and, thus, we ensured our pipeline could be improved iteratively and automatically if novel reactions are added to the BiGG database. Additionally, this pipeline enables us to propagate information (i.e. new reactions) from any one individual member of ParaDIGM to the rest of the collection of reconstructions.

10. Also, it was not clear to me whether the authors will regularly maintain and update the generated databases as new data arise or if this was more of a proposition that others can use the code in order to generate new models.

We are continuing to work on the models and codebase, but have made the code available for others to use (and contribute to) as well. This has been clarified on page 12 line 45.

Finally, in my opinion, an extremely interesting finding is also buried within the "Niche-specific metabolic function" paragraph and could be brought much more into play either in discussion or conclusion if the authors are confident enough, which is:
"Phylogeny is not the sole predictor of model similarity".
And I say this at the end because I believe that your findings support the idea that we should not just propagate ortholog reactions from the most similar species available because much of the information might be lost by doing this.

Thank you! We found this particularly intriguing as well and it serves as the basis for our follow on studies. It is highlighted in our abstract (page 1, line 20).


Minor comments:

0. I could not find table 1 within the material provided.

We erroneously omitted this table from our first submission, but included it in our resubmission. Please note, this table is now referenced as Table 2.

1. In Figure 2C, I would label the "unlabeled dots" or at least provide a subtitle in terms of which species each is referring to.

We have included this information in the caption for this figure.

2. Supplementary tables could have more information in tabs, column names and rows within the tables to improve readability. Also In Suppl. Table 4 I don't understand if the category "both" should be a sum of the other two (blocked/unconnected). If this is the case, I don't think the numbers add up.

We have included additional information into these tables on page 29 lines 25-29. The ‘both’ category is an intersection of the other two categories, or only the reactions that are present in both the ‘unconnected’ and ‘blocked’ categories. This is because we define ‘unconnected’ reactions as those whose *reactants* are not utilized by any other reactions and ‘blocked’ reactions as those whose *products* are not utilized by any other reactions (including transport and exchange reactions).

3. In Suppl Figures 2 and 3, the blue color circle on top of a purple color does give a good contrast to interpretation.

We removed color from these figures for clarity.

4. In Suppl Figure 3A: Do you see different trends from distinct databases regarding the number of reactions added at each step or is this very similar to all groups?

This analysis has only been performed for the species on PlasmoDB; future work will expand it to leverage the manual curation of other reconstructions to semi-curate other groups of organisms.

4. Page 5 line 31: I believe the sentence ends abruptly and is missing some words.

We have reread all the text around this point and are unable to locate an incomplete sentence.

5. In Figure 3E you could indicate from which database the species having the 7 metabolic unique reactions are coming from for easier readability.

This information has been added to Figure 3E (now Figure 4).

6. In general I don't see the point of including "data not shown", so if it's not relevant to your paper, I would just exclude it, otherwise please support the information necessary for this statement.

This phrase has been removed from the manuscript.

7. Page 7, line 4: "Most important variables" are variables here related to "reactions"?

The reviewer is correct – we have clarified this on page 8 line 23.

8. In page 10: you created the reconstructions for all "but nine" organisms. Which are these nine organisms? And why were they excluded? I found the explanation for T. gondii RH, but could not find other examples of excluded genomes.

We have clarified this statement – nine organisms have previously existing reconstructions so ours are the first iteration for all but nine of these organisms.

____________________________________________________________________________

Reviewer #2: This is a timely and important database. The paper is well-written. I have a few comments below, one of them more pressing, on the importance of transporter annotations.

The Introduction is very good at presenting the problem and need for this study, but it lacks a bit in presenting the state of the art for similar databases of GSMs-genome-scale models-and large-scale batch reconstruction efforts (e.g. ModelSEED, CarveME, BiGG, etc), as well as for large-scale comparative works with GSMs (mostly done in the aforementioned automated reconstruction tools, or evaluation tools e.g. MEMOTE). Comparisons of curated GSMs are very few, and use few models, (e.g. Xavier et al. Plos Comp Bio 2018) which highlights the need for databases as this one. I would advise a new paragraph before the last of the Introduction to do this - the authors already do mention much of this literature throughout the rest of the paper. In theory, the community as a whole would benefit from one, centralized source of good GSM data, (e.g. as done for genome data with NCBI), yet new ones continue to appear. Why is ParaDIGM important in this scenario, and how does it not increase division? This can be returned to in the Discussion as well. The usage of MEMOTE and BiGG nomenclature by the authors is commendable, and shows concern for community standards.

We have included a paragraph in the introduction to better summarize the state of the field and referenced this potential for division in the discussion section, summarized below:

Introduction (page 4 lines 7-18): Genome-scale metabolic models are built from genomic data and by inferring function to complete or connect metabolic pathways; these models are supplemented with data from functional genetic and biochemical studies, representing our best understanding of an organism’s biochemistry and cellular biology. Unfortunately, existing approaches for the construction of metabolic network models are lacking in standardization and scalability and/or biological relevance for eukaryotes. While there are pipelines that include compartmentalization (i.e. RAVEN and merlin (Agren et al. 2013; Dias et al. 2015)), individual high-quality parasite reconstructions (e.g. Carey, Papin, and Guler 2017; Abdel-Haleem et al. 2018; Chiappino-Pepe et al. 2017; Tymoshenko et al. 2015), and scalable pipelines for the construction of many networks (i.e. CarveMe, ModelSEED (Machado et al. 2018; Seaver et al. 2021)), we sought to build on these tools and the Eukaryotic Pathogens Database (EuPathDB, Amos et al. 2021) to leverage genomic information on the EuPathDB database and existing effort towards manual curation of individual reconstructions.

Discussion (page 14 line 11 onwards): The metabolic capacity represented in ParaDIGM will be expanded and the accuracy of each reconstruction and associated simulations will be improved as (1) BiGG is further expanded, (2) more confidence is gained regarding protein localization, and (3) metabolomics analyses improve biomass and media formulation. Additionally, we used our orthology-based semi-curation approach for only *Plasmodium* models; however, this approach can be used for other organisms to propagate manual curation efforts (from our group and others, e.g. Abdel-Haleem et al. 2018; Chiappino-Pepe et al. 2017; Tymoshenko et al. 2015) from one species to closely-related organisms as well.

Fig. 1A is more of a table than a figure. I think for the community it would be more helpful as a table, annotated with the references that convey the information given. To keep it as a figure though, it would really help to ditch the current grey color that does not convey information, and perhaps color code "yes" and "no"; it would also really help to sort the table, perhaps by the last column, perhaps split it by disease (which could come in a first column, non-repeated).

Figure 1A has been moved to a table (now Table 1) and sorted by disease.

How was KEGG information mapped to BiGG? Where is this mapping available for review? Please detail in the methods.

This has been clarified in the Methods section on page 16. In brief, nearly all BiGG identifiers have analogous KEGG identifiers. We downloaded the BiGG metabolites database (http://bigg.ucsd.edu/static/namespace/bigg_models_metabolites.txt) and used this mapping.

The most sensitive point: could you please expand on the process of annotation of transporters in the methods and discussion, perhaps consider a small analysis on how many were annotated per genome, and how many added through gap-filling?

We clarify on page 16 line 27 that transporters and enzymes are annotated using the same process, a Diamond-based annotation of all open reading frames against proteins in the BiGG database. Please see our new table Supplemental Tables 2 & 3 for a list of transporters in each reconstruction. Supplemental Table 2 shows the transporters annotated in each genome and Supplemental Table 3 shows which additional transporters were added via gapfilling.

In summary, approximately 53% of (intracellular and extracellular) transporters are genetically supported (see figure below, added as Supplemental Figure 8). *C. velia* CCMP2878 had the most genetically supported set of transporters (71.4%) whereas *T. cruzi* CL Brener had the least (21.3%). Please note, the *T. gondii* RH reconstruction was built from a partial genome (discussed on page 7 lines 16) and it represents the genome with 0% of transporters with gene annotations on the figure below.

These have a huge influence in GSMs, but in the case of parasites it becomes extreme (more so if one considers transport between compartments, important in the case of eukaryotes as the authors highlight). So as the authors recognize particularly with the correlation of some results with the environment of each species (Fig.4) transport will have a major impact in this work. KEGG does not have transporter annotations in the form of reactions, so they must come from Diamond-BiGG + Gapfilling, but how much of each? Being very explicit about this point will increase the value of ParaDIGM substantially.

We agree with all of the reviewer’s points - transporters are extremely important in compartmentalized reconstructions! We have added Supplemental Tables 2 & 3 and a discussion of this point in our limitations section (page 14 lines 3-4). We have also emphasized on page 29 in the table captions that transporters are only present if annotated in the genome or if required for a parsimony-based gapfilling solution. That being said, we are likely significantly under-annotating transporters.

In general the Figures are too crowded, with much text and schematics - things that would be better as boxes, tables or supplementary material. Some colors are undefined in the figures themselves; some figures are just crowded with gridlines etc., e.g. fig 5C the grey squares are confusing as the grey color is part of the key. This also occurs in Supplementary Figures, e.g. Supp Fig 5 - there's an excess of color and data and it's hard to discern what one can infer from this figure. Please review the figures in general for readability, consistency, and to remove excess content.

Thank you for this feedback. We have modified all of the figures to simplify color, improve consistency and remove excess content. You will notice that there are now only two color schemes being used throughout the manuscript – one to indicate database (introduced in Figure 1 and used in Figures 2-5 & 7 and Supplemental Figures 4-6) and one to indicate metabolite consumption/production (used in Figure 6 and Supplemental Figure 5). Color has been removed or turned to greyscale for all other figures. Regarding Supplemental Figure 5, we have clarified that our intent with this figure was not to communicate exactly which metabolites were being produced or consumed in each reconstruction but rather to communicate the sheer volume of data that can be obtained from ParaDIGM (page 9 line 46).

Lines 19-26 page 11 - the authors mean 4D instead of 4C, twice, I believe

This typo has been corrected.

Extensive work required on reference formatting; some names in format "Last, First" others in "First Last", urls heterogeneous, etc.

We appreciate the reviewer’s thoughtful read of the manuscript and will work with the editorial staff to standardize reference formatting if our manuscript is accepted for publication.

____________________________________________________________________________

Reviewer #3: 1)“Reaction compartmentalization was adjusted to maintain each gene-protein-reaction mapping but only with the subcellular compartments relevant for each organism. A large proportion of parasite gene-reaction pairs would otherwise be misassigned or removed from the network due to assignment to an incorrect compartment, due to lack of orthologous and compartmentalized reactions in biochemical databases; our pipeline reassigns these reactions to the cytosol (Figure 2B).”

-This should be explained more clearly as far as the” adjustments” that are happening. To be sure, does Figure 2B show only the percentage of reactions assigned to the cytosol? When I look at the legend below, I see that adjustment from the periplasm to extracellular is mentioned, not just cytosol re-assignments.

We clarified in the results section that these reactions are reassigned to the cytosol or extracellular space (page 5 line 21); this text previously had incorrectly just said ‘cytosol’ rather than ‘cytosol or extracellular space’. The process is also (and accurately) described on page 17.

2)“B): Considering compartmentalization. Our approach moves a large proportion of the reconstruction’s reactions from compartments in a biochemical database to biologically-relevant compartments (e.g. periplasm to extracellular)."

- “biologically-relevant compartments (e.g. periplasm to extracellular).”Is the periplasm not a biological-relevant compartment? Although I understand why to assign periplasm to extracellular as an abstraction, I don’t understand how the point being made about compartments in a database vs biologically-relevant compartments in the context of the example provided.

In eukaryotic organisms, the periplasm does not exist. We define biologically-relevant compartments as those that have been characterized in each respective organism. For example, the apicoplast is relevant in *Plasmodium* species but not most other organisms such as *Trypanosoma, Entamoeba*, or *Giardia*. Biologically relevant compartments are outlined in Supplemental Table 5.

-It is excellent that the authors are accounting for compartmentalization as they mentioned “, our de novo reconstruction approach accounts for compartmentalization, unlike previous metabolic network reconstruction pipelines.” I am unsure that the statement is correct. I believe that at least the metabolic reconstruction pipeline “merlin” accounts for compartmentalization as well (unsure about other tools that might have been released more recently).

-A little more clarification/changes in the paragraph text and legend would be appreciated to clarify this.

We clarified the sentence referenced above (added ‘unlike *many* previous metabolic network reconstruction pipelines’, page 5 line 37), expand our discussion in the Methods on page 17 line 42, and included a summary of the field on page 4 lines 7-18 that includes additional references including to merlin.

-Also, I only see the “pros” of making this compartment adjustment; as the authors mention, “large proportion of parasite gene-reaction pairs would otherwise be misassigned or removed”. What about the cons of doing this re-adjustment of reaction compartments and keeping those reactions?

Cons of this re-adjustment of reaction compartments include (1) we may move a compartment to the wrong compartment (i.e. it may not occur in the cytoplasm of the organism) and (2) the generation of new reaction IDs not yet in BiGG. However, the pros outweigh these cons; it would be inaccurate to keep the original reactions because this would result in (for example) the inclusion of a mitochondrial compartment in *Cryptosporidium* or the periplasm in any of these reconstructions.

3)"(D): Prediction accuracy. Semi-curated reconstructions (filled squares and triangles)"

-In the figure legend, filled squares are shown as "uncurated".

Thank you for pointing this out – it has been corrected in this resubmission.

4)“semi-curated reconstructions 8 are larger in scope than de novo reconstructions (Figure 2C) and generate predictions with comparable or improved accuracy (Figure 2D).”

-What does the increase in scope provide? What are we gaining from all those "extra" reactions when we only get "comparable accuracy" in the models?

Gene essentiality datasets are collected from only one or two life stages for each parasite, whereas the reconstructions represent all metabolism, regardless of stage. As experimental (validation) data is collected from more life stages, models larger in scope can be tested against a larger fraction of these data.

I looked at the supplementary material data to see if this increase in the number of reactions also meant an increase in the number of blocked reactions in the model but could not find out.

The mean number of blocked reactions in each reconstruction before gapfilling is 18 and after gapfilling is 19. The mean number of unconnected reactions before gapfilling is 38 and after gapfilling is 31. Lastly, the mean number of reactions that are both blocked and unconnected before and after gapfilling is 6. See distributions below, now in Supplemental Figure 9.

5)"The relationship between genome size and model size is weakened following gapfilling (Supplemental Figure 4D)"

-Can you explain why this happens? I guess that biomass requirements/biomass equation will be the same for many of these draft reconstructions (since accurate data on this is limited), meaning gap-filling will add a lot of the same reactions to fulfill the exact biomass requirements.

We agree with the reviewer’s hypothesis and have added it to the text on pages 7-8 lines 49-1.

6)Page 8, line 23 "Interestingly, several metabolic enzymes were consistently predicted to be necessary for observed metabolic capabilities (Figure 5C) or growth across all parasites (Table 1)"

-I am trying to understand how Figure 5C supports this claim along with Table 1. But it seems that Table 1 is missing. I can only find a legend for it: “Table 1: Most frequently gapfilled reactions. These reactions (in the BiGG namespace) were the most commonly added reactions as a result of all gapfilling steps.”
Either table 1 missing, or does it refer to supplementary table 1, and do we have to parse the data from there to understand what is claimed here?

Table 1 was missing from our first submission – we apologize and include it in our resubmission. Please note, it is now referenced as Table 2. Additionally, we have clarified our references to these figures:

Page 10 lines 1-3: "Interestingly, several metabolic enzymes were consistently predicted to be necessary for observed metabolic capabilities (metabolic tasks in Figure 6C) or growth across all parasites (gapfilled reactions in Table 2)."

7)"While reaction essentiality is generally more similar for closely related organisms (i.e. within genera), essentiality predictions were more similiar when comparing Plasmodium genomes to one another than between Toxoplasma or Cryptosporidium genomes.”

-I am confused here. Aren’t the results showing that as expected (“generally”) essentiality results are more similar for closely related Plasmodium genomes. I guess my confusion stems from the phrase starting with “while”, I assumed that the conclusion would be otherwise to what “generally” happens. So it’s either that or I am interpreting Figure 6 wrong.

We have reworded this sentence for improved clarity as follows:

Page 12 lines 9-12: “Reaction essentially is generally more similar for closely related organisms (i.e. within genera). However, some genera generate more similar predictions than others. Essentially predictions were more similar when comparing *Plasmodium* genomes to one another than between *Toxoplasma* genomes, despite all genomes being of the same species, or *Cryptosporidium* genomes.”

I want to compliment the authors on clearly explaining the critical limitations of their pipeline in the discussion session. That addressed multiple questions I was going to include in my review.

Thank you!
